# Testing for selection bias and moral hazard in private health insurance: Evidence from a mixed public‐private health system

**DOI:** 10.1002/hec.4605

**Published:** 2022-09-13

**Authors:** Clifford Afoakwah, Joshua Byrnes, Paul Scuffham, Son Nghiem

**Affiliations:** ^1^ Centre for Applied Health Economics Griffith University Queensland Nathan Australia; ^2^ Menzies Health Institute Queensland Griffith University Queensland Southport Australia; ^3^ College of Health & Medicine Australian National University Canberra Australia

**Keywords:** cardiovascular disease, moral hazard, private health insurance, selection bias

## Abstract

Separating selection bias from moral hazard in private health insurance (PHI) markets has been a challenging task. We estimate selection bias and moral hazard in Australia's mixed public‐private health system, where PHI premiums are community‐rated rather than risk‐rated. Using longitudinal cohort data, with fine‐grained measures for medical services predominantly funded by PHI providers, we find consistent and robust estimates of advantageous selection among hospitalized cardiovascular disease (CVD) patients. Specifically, we show that in addition to their risk‐averse attributes, CVD patients who purchase PHI use fewer services that are not covered by PHI providers (e.g., general practitioners and emergency departments) and have fewer comorbidities. Finally, unlike previous studies, we show that *ex‐post* moral hazard exists in the use of specific “in‐hospital” medical services such as specialist and physician services, miscellaneous diagnostic procedures, and therapeutic treatments. From the perspective of PHI providers, the annual cost of moral hazard translates to a lower bound estimate of $707 per patient, equivalent to a 3.03% reduction in their annual profits.

## 1 INTRODUCTION

The Australian health insurance market is characterized by a mixed system of universal public health insurance, Medicare, and a subsidized private health insurance (PHI) market. Medicare is comprehensive and covers a significant range of medical services (van Gool et al., 2015), all of which are listed in the Medicare Benefits Schedule (MBS). While all Australians are supported by Medicare, individuals have strong incentives to purchase PHI. First, these include access to more health cover and choices. PHI allows patients to access health services that are not covered by Medicare (e.g., through top‐up extras covering physiotherapy, dental or optical) and choose their preferred doctors, hospital (public or private), and treatment scheduling. Although patients without PHI have access to these services, they must cover the out‐of‐pocket costs. Second, PHI patients are able to circumvent waiting times for planned surgery and quickly access hospital procedures that are not medical emergencies (elective procedures). Third, PHI patients are exempted from the government's Medicare Levy Surcharge (MLS), an additional income tax payable by individuals without PHI. The MLS aims to incentivize the purchase of private hospital cover to reduce the demand for public hospital services. Fourth, patients with PHI may also receive PHI rebates in the form of lower PHI premiums or an annual tax return offset. Finally, those who purchase PHI at a young age are able to avoid paying higher PHI premiums as they age (Australian Government Department of Health and Aged Care, 2021; Buchmueller et al., 2013; BUPA, 2022).

Beyond these benefits, PHI premiums are also not risk‐rated in that PHI providers are prohibited from using individuals' observable characteristics to set premiums. Considering the unique nature of the Australian PHI market, this study investigates the possibility of selection bias and moral hazard in the PHI market using longitudinal population‐based cohort data of people living with cardiovascular diseases.

At market equilibrium, a positive correlation should exist between individual risk levels and the uptake of PHI (Chiappori et al., 2006). That is, in a “community‐rated” PHI market like Australia, adverse selection will more likely prevail. Adverse selection is the situation whereby people who have greater health risk are more likely than their counterparts to purchase health insurance (Akerlof, 1970; Rothschild & Stiglitz, 1976; Spence & Zeckhauser, 1971). This is because those at high risk know that their expected healthcare spending is high, and that PHI will lower their out‐of‐pocket expenditure (Akerlof, 1970; Rothschild & Stiglitz, 1976). Conversely, low‐risk individuals may purchase PHI for its advantages. This type of selection bias is known as advantageous selection. As such, selection bias in the PHI market could impact the health insurance provider's ability to cover its cost. Previous studies on the uptake of PHI in the Australian market are mixed; some found adverse selection (Barrett & Conlon, 2003; Nguyen & Worthington, 2017; Savage & Wright, 2003), while others noted advantageous selection (Buchmueller et al., 2013; Nghiem & Graves, 2019).

Further, ownership of PHI can lead to moral hazard due to asymmetric information where the insurer cannot observe the change in behavior of the insured (*ex‐ante*). In the presence of moral hazard, privately insured individuals take more health risks knowing that they will not bear the full cost of the consequences and tend to overconsume health resources than those who are uninsured. Another form of moral hazard in the PHI market occurs when those who are insured over‐consume healthcare services beyond the quantities they would require if they did not have PHI (*ex‐post*) (Pauly, 1968). In both cases, moral hazard leads to an increase in the cost of health care, and thus, greater cost to the insurance provider (Doiron et al., 2013).

Studies on the Australian PHI market examined selection bias and moral hazard using data on the general population, mostly from the Australian Bureau of Statistics (ABS) (Buchmueller et al., 2013; Eldridge et al., 2017). While such data reveal the extent of selection bias and moral hazard in the population, they do not capture the heterogeneous behaviors among those with different health conditions. People with chronic health conditions such as cardiovascular disease (CVD), diabetes and cancer may prefer to purchase PHI due to their frequent need for healthcare services. Such chronic conditions require treatment and management of recurring episodes throughout the life‐cycle of the disease, which imposes a significant burden on the affected families. For example, in Australia, it is estimated that a “simple” CVD hospitalization costs $2477 (Afoakwah et al., 2020) while a complex hospitalization costs around $7260, with a monthly out‐of‐pocket cost of $258 for treating and managing the condition (Saunders, 2018). Hypothetically, considering the high cost of the disease coupled with the frequent demand for healthcare services, people living with CVD will be more likely to purchase PHI knowing that their expected healthcare cost will be higher than the premium they pay for PHI.

In this study, we investigate the impact of asymmetric information regarding individuals' CVD risk factors on the uptake of PHI leading to adverse selection (high‐risk individuals being more likely to purchase PHI) or advantageous selection (low‐risk individuals being more likely to purchase PHI) and moral hazard (higher consumption of health services by those with PHI) among people living with CVD in Australia. We focus on a population of people living with CVD to re‐examine the correlation between risk level and PHI uptake. Our study focuses on all CVD patients who were first hospitalized with any CVD condition in Queensland, Australia, during the 2010 calendar year. These patients were followed until December 2015 with detailed information collected on their use of in‐hospital healthcare services (e.g., hospitalizations, specialists, physicians, miscellaneous diagnostics and therapeutic procedures) as well as their out‐of‐hospital healthcare service usage (e.g., general practitioner (GP) visits). Data on their emergency department (ED) presentations were also collected. Using this detailed information on their use of primary and secondary healthcare services coupled with the unique nature of the Australian PHI market, we provide consistent and robust estimates of selection bias and moral hazard among hospitalized CVD patients.

In doing so, we make three contributions to the literature. First, we explore selection bias in the uptake of PHI from different perspectives. Specifically, beyond examining the socio‐demographic and economic attributes that characterize the uptake of PHI, we explore the use of medical services that are not funded by PHI providers (such as GP services). This setting presents a quasi‐experiment to reveal the health risks associated with those with PHI since moral hazard in the use of such services is not expected. In addition to the use of GP services, we also compare the use of ED services between those with and without PHI. While ED visits are dependent on the severity of the presentation and do not require appointments or long waiting times like elective procedures, frequent ED presentations demonstrate certain unobservable health and socio‐demographic characteristics of patients. Markham and Graudins (2011) found that relative to non‐frequent ED presenters, those who frequently present to EDs in Australia are more likely to arrive in police custody, be admitted to a mental health bed and/or to self‐discharge while awaiting care. To further reveal the unobservable health risk (selection bias) in the uptake of PHI, we analyze the differences in survival time and frequency of multiple‐day hospital episodes between those with and without PHI. Therefore, by examining selection bias from different angles, our study provides robust and reliable estimates of selection bias in the Australian PHI market. These findings will serve as a benchmark to determine whether the Australian Government's strategy of using an insurance premium surcharge to encourage PHI uptake leads to selection bias in the PHI market.

Second, we demonstrate that selection bias and moral hazard in PHI can be separately estimated by exploiting unique characteristics of the Australian PHI market and record linkage hospital data. In this study, we use rich longitudinal population‐based cohort data, with fine‐grained measures for healthcare services that are largely funded by PHI providers, to estimate moral hazard. Our detailed hospital and MBS data allow us to analyze the types of care received in hospital, thereby unpacking the different procedures that are important sources of payments for PHI providers. Specifically, we focus on moral hazard in the use of expensive cardiac healthcare services such as specialists, physicians, and miscellaneous diagnostic (e.g., 12‐lead electrocardiogram) and therapeutic procedures (e.g., foreign body removal, superficial wound closure, small abscess or pul space drainage, removal of nail of finger/thumb/toe). Previous studies focused on only estimating moral hazard in hospital admissions. While hospital admissions alone reflect the use of healthcare services in a heterogeneous population, they may not capture the true spectrum of healthcare services consumed by chronically ill people. For example, the use of same‐day hospital services may not be captured by hospital admissions data but still presents a substantial cost burden to the health sector and PHI providers. Our rich data allow us to isolate moral hazard in the use of these services separate from the traditionally used hospital admissions.

Third, we estimate costs associated with moral hazard from the perspective of PHI providers. Specifically, we estimate how much the differences in the use of healthcare services between those with and without PHI cost the PHI provider. Such estimates are useful for determining the equilibrium premiums that can compensate for the excessive use of healthcare services among those with PHI.

Our study is structured as follows. Section 2 presents background information on the Australian PHI market and previous studies. In Sections 3 and 4, we discuss the methods and data, respectively, which is followed by presentations of results in Section 5. Section 6 reports robustness checks and additional results, which are followed by a discussion of the key findings and concluding remarks in Section 7.

## 2 BACKGROUND

### 2.1 The Australian health system and PHI

Australia has one of the most comprehensive health care financing models in the world. The public Medicare program was established in 1984 and covers more than 5700 different medical services, which are delivered both in‐ and out‐of‐hospital by either private or public health care providers (van Gool et al., 2015).

**FIGURE A1:**
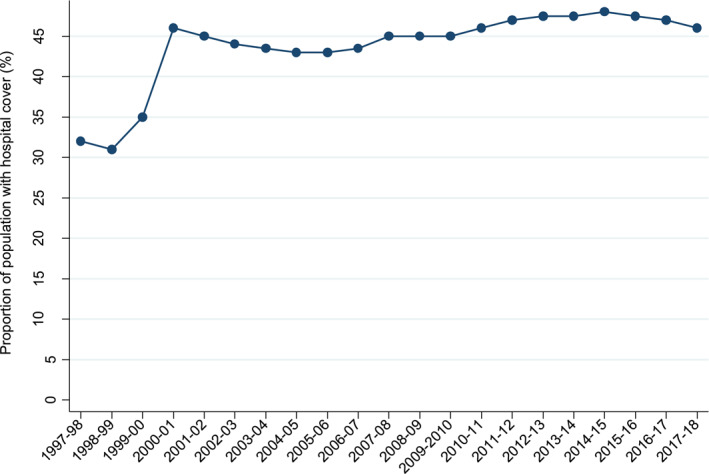
Private health insurance coverage in Australia, 1997‐98 to 2017‐18. Membership is reported in calendar years for hospital cover. *Source*: (AIHW 2019b); APRA 2019

Despite the comprehensive nature of Medicare, a substantial proportion of Australians still purchase PHI. According to the Australian Prudential Regulatory Authority (APRA), 44.2% of Australians had private hospital cover as of March 31, 2021 while the total number of insurance scheme members who had coverage for hospital treatment increased by 170,219 for the same period relative to the previous year (Australian Prudential Regulatory Authority, 2021). Figure A1 shows that PHI coverage has been stable since 2000. That is, people purchase PHI for the five key benefits: having access to higher cover and more choice, avoiding queues for planned surgery, being exempted from the MLS, accessing additional tax offsets and/or reducing the cost of future PHI premiums during old age. That is, beyond individual‐level characteristics such as health risk and risk‐taking behaviors that often drive selection into PHI, government policies such as the MLS can also generate selection bias depending on individual income levels.

One important feature of the Australian health system is that it is a mixed public and private system, whereby a patient who has PHI can choose to be treated at a public hospital as a private patient, in which case the cost of the service will be covered by the PHI provider. Conversely, a patient without PHI may sometimes be assigned to receive care at a private hospital when there are extremely long waiting times at public hospitals; in this case, the service is covered by Medicare. What is notable is that the decision of whether a patient is public or private is made at admission and the two systems do not “stack”; a patient cannot be admitted as a public patient to a public hospital and then initiate the use of their PHI to “upgrade” their care. For example, during the 2017–18 financial year, 13% of hospitalizations in public hospitals were funded by PHI, while 83% of hospitalizations in private hospitals were funded by PHI (Australian Institute of Health and Welfare, 2020b).

PHI premiums in Australia are “community‐rated” rather than “risk‐rated”, meaning that PHI providers cannot set individual premiums based on the observable characteristics of a person, nor can they exclude patients based on pre‐existing conditions. This leads to information asymmetry in the PHI market. In addition to the community‐rated premium system, the *Private Health Insurance Act 2007* prohibits PHI providers from offering cover for out‐of‐hospital services that are otherwise funded by Medicare, such as GP visits, consultations with specialists in their private rooms and out‐of‐hospital diagnostics (Australian Government Department of Health, 2019). Hence, we can analyze the differences in the use of out‐of‐hospital services between those with and without PHI to reveal the type of selection bias. Similarly, differences in the use of in‐hospital services between those with and without PHI reveal moral hazard in PHI.

### 2.2 Previous research

The theoretical underpinning of this study is centered on earlier models of insurance by Arrow (1963) and Pauly (1974). This was further developed by Rothschild and Stiglitz (1976) with a key assumption that individuals who purchase PHI have one‐dimensional private information regarding their own health risk, leading to an ex‐ante adverse selection. Adverse selection implies that people who are less healthy are more willing to purchase PHI because they expect their healthcare spending to be high (Akerlof, 1970; Rothschild & Stiglitz, 1976). Market equilibria are likely to be characterized by a positive correlation between risk and the level of insurance coverage, driving low‐risk individuals away from the market. However, accounting for heterogeneity in risk preferences can generate different results about risk selection. For example, if risk‐averse individuals are both more likely to purchase PHI and more likely to make efforts to reduce the risk of experiencing a loss after the insurance contract has been activated, then adverse selection may not hold; rather, advantageous selection will prevail (Hemenway, 1990, 1992). The presence of post‐contractual asymmetric information in health insurance markets can also result in the insured taking less preventative action to avoid loss (*ex‐ante* moral hazard) or over‐consuming healthcare services (*ex‐post* moral hazard). In the context of CVD patients, moral hazard (if any) would be ex‐post; that is, patients seeking extensive diagnosis and treatment after the PHI contract has been activated. The presence of selection bias or moral hazard leads to market failure.

Previous studies explored the presence of selection bias and moral hazard in different markets, and the findings are mixed. In annuity markets, a positive correlation between insurance uptake and loss occurrence has been established (Finkelstein & Poterba, 2002, 2004; Mitchell & McCarthy, 2002). Contrarily, no evidence of positive correlation was found in the automobile (Chiappori & Salanie, 2000; Dionne et al., 2001), life insurance (Cawley & Philipson, 1999), and long‐term care insurance markets (Finkelstein & McGarry, 2006). In the PHI market, evidence of negative correlation (advantageous selection) was noted in the US (De Meza & Webb, 2001; Fang et al., 2008), with multidimensional asymmetric information detected in Ireland (Bolhaar et al., 2012). Keane and Stavrunova (2016) analyzed two different data sets from the US, Medicare Current Beneficiary Survey and Health and Retirement Study, and found evidence of advantageous selection into Medigap when they controlled for only Medigap pricing variables. However, they found that adverse selection into Medigap prevailed when they further control for individuals' private information variables in their model. The authors further estimated a substantial amount of moral hazard that is equivalent of US$1615 in healthcare expenditure. In summary, evidence from the PHI markets in different countries is mixed and largely dependent on the nature of the country's PHI market and the modeling approaches.

In the Australian PHI market, previous studies found mixed results on the correlation between PHI uptake and the occurrence of loss. Nghiem and Graves (2019) analyzed administrative data of skin cancer patients and found evidence of advantageous selection in the uptake of PHI. Specifically, they observed that patients with PHI were more likely to take protective measures such as applying sunscreen or wearing a hat outdoors to mitigate the aggravating effects of chronic exposure to ultraviolet sunlight. However, they also found that patients with PHI had higher healthcare expenditures than those without PHI, suggesting an *ex‐post* moral hazard. Similarly, Buchmueller et al. (2013) conducted a comprehensive study on the correlation between health risk and PHI uptake in the Australian PHI market. After carefully analyzing data from the Australian National Health Survey, they found that risky individuals were less likely to purchase PHI. In addition, they noted that individuals with PHI used fewer GP services and spent fewer nights in the hospital. That is, risk aversion explains the advantageous selection in the Australian PHI market. Similarly, Eldridge et al. (2017) analyzed data from the Australian National Health Survey and found that ownership of PHI was positively associated with odds of hospital admission as a private patient.

It is important to highlight that none of the previous studies investigated moral hazard in the use of healthcare services that are largely funded by PHI providers (such as the use of specialist and physician services, diagnostics, and therapeutic procedures). Detailed analysis of medical services that constitute the benefits paid by PHI providers will better unpack the extent of moral hazard in the health insurance market. In this study, we link hospital data with the MBS to first examine the correlation between health risk and the update of PHI. We proceed to produce, for the first time, estimates for moral hazard in the use of healthcare services that are substantially funded by PHI in a mixed public‐private healthcare system. We exploit longitudinal population‐based cohort data of CVD patients who had their first CVD hospitalization in 2010, with subsequent hospitalizations until December 2015. The hospital data are linked with MBS data, which have fine‐grained measures for healthcare services that are largely funded by PHI providers in Australia. The longitudinal nature of our data allows us to track individual health conditions to predict patients' uptake of PHI and their use of healthcare services over time.

## 3 EMPIRICAL STRATEGY

### 3.1 Selection bias

We can investigate the correlation between health risk and PHI uptake by modeling demand for PHI using the expected utility hypothesis. That is, an individual will purchase PHI if his/her expected indirect utility with PHI (${PI}^{*}$) is greater than that without PHI. Therefore, the decision to purchase PHI is specified as:

(1) $${PI}_{i,s,t}^{*}=\varphi +\omega X_{i,s,t}{+\rho_{s}+\tau_{t}+\varepsilon }_{i,s,t}$$

where ${PI}^{*}$ is associated with the observed PHI (*PI*) of individual *i* in geographic location *s* at year *t*, such that PI=1 if ${PI}^{*}\geq 0$ and where PI=0 otherwise. *X* is a vector of observable covariates, including individual characteristics, that influence the decision to purchase PHI. The vector *X* includes a mixture of binary variables (e.g., sex, ethnicity, marital status) and categorical variables (e.g., age group). Since hospitals do not collect information on patients' economic status (e.g., income), we control for this important variable by including a categorical variable for the socioeconomic status of the patient's postcode of usual residence using data on Socio‐Economic Indexes for Areas (SEIFA) from the ABS. The ABS generates the SEIFA index from a range of variables, including income, employment, occupation, education, and housing characteristics. Here, SEIFA is presented in quintiles (Q) ranging from Q1 (lowest socioeconomic status) to Q5 (highest socioeconomic status). We also control for the unemployment rate across geographic locations by linking our hospital data with unemployment data from the ABS. Geographic locations are defined using Statistical Area Level 4s (SA4s). SA4s are the largest sub‐state regions in Australia and their boundaries represent labor markets and the functional areas of capital cities. The location of this study, the state of Queensland, is divided into 19 SA4s (Australian Bureau of Statistics, 2021).^1^ The health risk is proxied by the comorbidity status of the individual. Specifically, we use the Charlson Comorbidity Index (CCI), which captures the severity of a person's condition at hospital presentation. CCI was computed from our data based on only secondary diagnosis codes with an onset flag of “present on admission”. Finally, *ρ* and *τ* are SA4 and year fixed effects, and ε is the error term.

If adverse selection prevails in the PHI market, we expect that CVD patients with a high health risk will most likely purchase PHI. Such health risk includes old age, low socioeconomic status, and high comorbidities. Assuming that $\varepsilon_{i,s,t}$ is normally distributed, Equation (1) can be estimated using a Probit regression (Cameron & Trivedi, 2005). Equation (1) is often estimated with a restrictive assumption that the error term $\varepsilon_{i,s,t}$ and covariates in *X* are independently and identically distributed (IID), which can be empirically challenging. For example, error terms, which capture unobserved individual and regional characteristics, could be dependent within a cluster (i.e., postcodes or SA4s), and thus, violate the IID assumption. We applied the heteroskedasticity robust standard errors by White (1980) to produce standard errors that have valid statistical confidence intervals of the estimates even if the error term is not independently distributed (i.e., the variance of the residuals may not be constant). This technique is also referred to as ‘sandwich standard errors’ because it is calculated by matrix multiplication of two original standard errors (i.e., bread) and the weighted sum contribution to the total variance (i.e., the sandwich filling) of each observation (or each cluster if cluster sampling is available and the assumption of the dependent distribution of the residual within a cluster is valid). Thus, standard errors calculated using the sandwich method are robust where the variance of the residuals is not constant (i.e., where heteroskedasticity exists).

Due to the community rating system in Australia, which prevents PHI providers from setting premiums based on individuals' observable characteristics, another natural means of estimating adverse or advantageous selection is by analyzing the differences between those with and without PHI in terms of their use of healthcare services not funded by PHI providers. Specifically, in the setting of the Australian health system, we can examine the differences in the number of GP visits and ED presentations between those with and without PHI to reveal the nature of selection bias in PHI uptake. In this setting, simply regressing GP visits and ED presentations on PHI status without any control will reveal the extent of selection bias (Buchmueller et al., 2013). We further modify the specification by including the control variables explained above in the models to demonstrate that the unobservable individual attributes do not compromise the correlation between health risk and PHI uptake. The Ordinary Least Squared (OLS) method is used to estimate the association between PHI status and the use of GP and ED services.

### 3.2 Moral hazard

The demand for healthcare services that are funded by PHI providers is specified as:

(2) $${HS}_{i,s,t}^{k}=\alpha {PI}_{i,s,t}+\gamma X_{i,s,t}+\rho_{s}+\tau_{t}+\mu_{i,s,t}$$

where ${HS}^{k}$ (*k =* 1, 2 … *K*) is a vector of healthcare services used by CVD patients that are covered by PHI in Australia. Elements of *HS* are in continuous data formats, and the OLS estimator for Equation (2) is used accordingly. In this study, we focus on five services including the number of hospital admissions, the number of specialist and consultant physician services, and the numbers of miscellaneous diagnostic and therapeutic procedures. PI, X, *ρ* and τ are defined as in Equation (1); μ is the random error term.

The decision to purchase PHI and use healthcare services may be determined by certain unobservable characteristics. That is, individuals purchase PHI for multiple reasons, including their level of health risk, which may not be observable to the PHI provider. These unobserved individual characteristics (e.g., health risk and risk attitudes) are included in the error term, leading to a potential correlation between *PI* and μ in Equation (2). Thus, applying standard estimators to Equation (2) could produce biased estimates.

We undertake several exercises to mitigate this potential bias. First, we control for a set of rich individual‐level health risk attributes, such as comorbidity and age, which have been shown to be risk factors for cardiovascular health. Comorbidity, for example, has been shown to be a high‐risk factor not only for CVDs but also for respiratory diseases such as the coronavirus disease of 2019 (COVID‐19) (Alam et al., 2021). Second, we control for community‐level (proxied with SA4s) time‐varying covariates such as economic activities and population growth by including variations in unemployment and the number of civilian members of the population aged 15 years and above across SA4s in our model. These observable time‐varying community‐level characteristics unpack the extent of risk among individuals in the same community, which is used to set premiums by PHI providers. Afoakwah et al. (2021) also showed that variations in the local level unemployment rate affect the demand for healthcare services among CVD patients. Third, we control for community and year fixed effects in the models. Community fixed effects capture unobservable time‐invariant characteristics across communities that can impact individuals' healthcare use but are not captured in our model. These community‐level variables are particularly important because they are used by PHI providers to set premiums. Also, the year fixed effects pick up changes in policies, technology or practices over time, which are beyond the patients' control but affect both their uptake of PHI and use of healthcare services. By doing this, we isolate any confounding effects across communities. Equation (2) is estimated using OLS because the five selected health services in the HS set are presented as continuous real numbers (see Table A2 for details). Although the number of healthcare services used can be considered as a count variable (in which case a Poisson maximum likelihood estimator would be appropriate), we follow previous work (Buchmueller et al., 2013; Liu et al., 2012) and apply the OLS estimator for ease of interpretation of the coefficients as moral hazard. We also estimate robust standard errors (White, 1980) to mitigate the effects of potential unknown heteroskedasticity.

To validate the robustness of our OLS estimates, we later use the propensity score matching and conditional‐mixed process estimators to re‐estimate the moral hazard in the use of healthcare services that are funded by PHI providers.

## 4 DATA AND DESCRIPTION OF KEY VARIABLES

### 4.1 Source

Data for our study are from the Queensland Cardiovascular Record Linkage (QCARD) dataset. QCARD is a linked longitudinal cohort dataset of people living with CVD from Queensland, Australia (Byrnes et al., 2020). The data include records of admission and ED presentations that are linked with data from the MBS. It includes 135,399 people who were hospitalized with CVD as a primary diagnosis in 2010. We focused on a subset of 84,821 patients who had their first CVD hospitalization in 2010 with follow‐up hospitalizations until December 2015. Hence, data on their use of in‐ and out‐of‐hospital healthcare services over this period were collected from MBS and linked with the hospital data. One limitation of this dataset is that it excludes CVD patients who were not hospitalized during the study period; hence the findings from this study should be interpreted with caution. The data linkage process is available in Byrnes et al. (2020).

### 4.2 Outcome variables

Since the focus of our study is to estimate selection bias and moral hazard in the PHI market, our outcome variables are the number of healthcare services used. This allows us to compare the number of services used by patients with PHI to those without PHI. To test for selection bias in the Australian PHI market, we use two variables that are not covered by PHI: GP visits and ED presentations. Since PHI in Australia does not cover the use of out‐of‐hospital physician services, the difference between the number of GP services used by patients with and without PHI reveals variations in health risk between the two groups (Buchmueller et al., 2013). Also, ED presentations do not have long waiting times for treatment relative to elective procedures and hence, their examination reveals the nature of the correlation between the patients' health risk level and the uptake of PHI.

**FIGURE A2:**
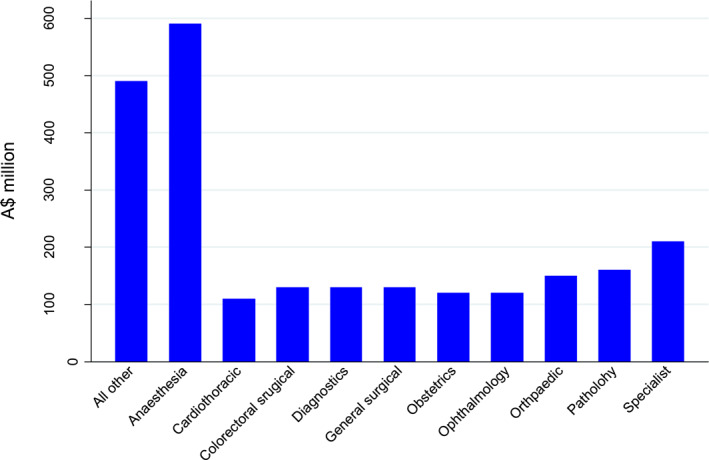
Benefits paid by private health insurers by service type and hospital treatment during 2018–2019 in Australia. *Source*: ARA 2019

With regards to moral hazard, we focus on in‐hospital medical services that are listed in the MBS and thus, can be covered by either Medicare or PHI. The MBS data include an MBS hospital flag, which is used to exclude out‐of‐hospital medical services from this analysis. Specifically, we focus on the number of hospitalizations, specialist and consultant physician services used, and miscellaneous diagnostic and therapeutic procedures. These variables are used to test for the presence of moral hazard because they constitute a substantial source of funding from the perspective of PHI providers, as shown in Figure A2.

### 4.3 Main independent variable

Our main independent variable is ownership of PHI. From the hospital database, we are able to identify patients who have PHI. Since our data are from the hospital, ownership of PHI implicitly means that it covers hospital care. This variable is captured as a dummy variable that equals one if the patient had PHI at all hospital admissions during the calendar year and otherwise equals zero. As a robustness check, we later redefine ownership of PHI as those who had PHI at least one hospital admission during the calendar year. A limitation of our proxy for PHI is that the specific coverage details (e.g., which treatment options are included/excluded) of patients' PHI policies are not known. Hence, we assume that PHI at hospital presentation also covers all secondary procedures.

## 5 RESULTS

### 5.1 Descriptive statistics

**FIGURE 1:**
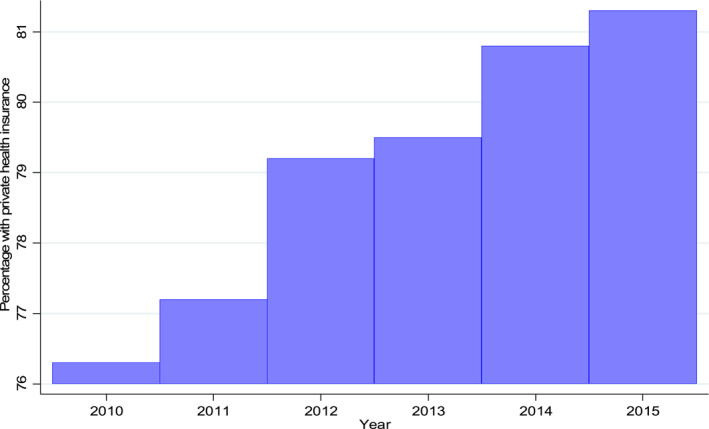
Private health insurance ownership overtime

Figure 1 shows that 76.3% of patients had PHI during the year of their first CVD hospitalization (i.e., in 2010). This percentage is substantially larger than the average 46% PHI uptake among Australians reported by the AIHW. Note, however, that our data cover older adults (67% of patients in our data are aged 65 years and above) who are chronically ill. The proportion of patients with PHI increased by 0.9% points after 1 year and by 5% points 5 years later. Table A3 shows that relative to those with PHI at their first CVD hospitalization, those without PHI at index hospitalization had a substantially higher probability of purchasing PHI thereafter. Table 1 shows a descriptive analysis of key variables used in this study.^2^ CVD patients with PHI had fewer GP visits, ED presentations and hospital admissions compared with those without PHI. However, patients with PHI had more in‐hospital specialist and physician services, miscellaneous diagnostics and therapeutic procedures compared to patients without PHI. The lower number of GP visits, ED presentations and hospital admissions (that are fully covered by Medicare) among patients with PHI suggest an advantageous selection. Further, the high usage of medical services that are largely funded by PHI providers suggests an *ex‐post* moral hazard in the PHI market. Accordingly, patients who had PHI were younger, mostly non‐Indigenous Australians, of high socioeconomic status, who were married and had fewer comorbidities than those without PHI. These characteristics of patients with PHI corroborate the finding of advantageous selection in the uptake of PHI in Australia.

**TABLE 1:** Descriptive statistics

Variables	With PHI	Without PHI	Difference
	*N* = 154,749	*N* = 43,324	
GP visits	12.769	14.379	−1.891***
ED visits	1.803	2.353	−0.550***
Hospital admissions	3.487	5.394	−1.908***
Specialist services	2.961	2.332	0.630***
Physician services	9.252	7.320	1.932***
Miscellaneous diagnostic procedure	2.419	1.782	0.637***
Therapeutic procedures	1.207	0.814	0.394***
Male	0.520	0.546	−0.026***
Indigenous	0.004	0.025	−0.021***
Age (years)			
18–44	0.058	0.071	−0.013***
45–64	0.286	0.219	0.067***
65 and above	0.656	0.710	−0.054***
Socioeconomic status			
Q1	0.127	0.191	−0.064***
Q2	0.162	0.190	−0.028***
Q3	0.207	0.200	0.007***
Q4	0.223	0.209	0.014***
Q5	0.282	0.211	0.071***
Marital status			
Divorced	0.057	0.070	−0.013***
Married/de facto	0.685	0.511	0.174***
Never married	0.089	0.138	−0.049***
Widowed	0.167	0.280	−0.113***
Comorbidity status			
No	0.454	0.318	0.136***
One	0.137	0.134	0.003
Two or more	0.408	0.548	−0.14***

Abbreviations: ED, emergency department; GP, general practitioner; PHI, private health insurance.

***p < 0.01

### 5.2 Selection bias

We now examine selection bias in the PHI market. Table 2 shows that males and those of Indigenous descent are significantly less likely to purchase PHI. The uptake of PHI is also positively associated with aging, with the highest uptake observed among those aged 45–64 years relative to the youngest adult group (18–44 years) and the aged (65 and above). Married individuals are most likely to purchase PHI compared to those divorced, never married, or widowed. Finally, we found that individuals of high socioeconomic status are more likely to purchase PHI, while relative to CVD patients with no comorbidities, those with one or more comorbidities were less likely to purchase PHI. These findings suggest advantageous selection in the uptake of PHI: that is, those with low health risk and of high socioeconomic status purchase PHI.

**TABLE 2:** Probit estimates for determinants of private health insurance uptake

	Coefficients	Marginal effects
Male	−0.170*** (0.007)	−0.046*** (0.002)
Indigenous	−0.953*** (0.031)	−0.261*** (0.009)
Age (years; ref = 18–44)		
45–64	0.295*** (0.015)	0.081*** (0.004)
65 and above	0.241*** (0.014)	0.066*** (0.004)
Marital status (ref = divorced)		
Married	0.328*** (0.013)	0.090*** (0.004)
Never married	−0.049*** (0.016)	−0.014*** (0.004)
Widowed	−0.176*** (0.015)	−0.048*** (0.004)
Socio‐economic status (ref = Q1)		
Q2	0.130*** (0.015)	0.036*** (0.004)
Q3	0.288*** (0.015)	0.079*** (0.004)
Q4	0.279*** (0.016)	0.077*** (0.004)
Q5	0.368*** (0.017)	0.101*** (0.005)
Comorbidity (ref = no comorbidity)		
1	−0.143*** (0.010)	−0.039*** (0.003)
2 and above	−0.309*** (0.008)	−0.085*** (0.002)
Year (ref: 2010)		
2011	0.077*** (0.010)	0.021**** (0.003)
2012	0.140*** (0.010)	0.039*** (0.003)
2013	0.139*** (0.011)	0.038*** (0.003)
2014	0.184*** (0.011)	0.050*** (0.003)
2015	0.190*** (0.011)	0.052*** (0.003)
SA4 fixed effects	Yes	Yes
*N*	198,073	
Pseudo *R* ^2^	0.067	

*Note*: Robust heteroskedasticity standard errors are in parenthesis. McFadden (1974) pseudo *R*‐squared is reported.

***p < 0.01, **p < 0.05, *p < 0.1.

In Australia, GP visits are not covered by PHI^3^ and ED presentations are fully covered by Medicare through the hospital costing system. These two services can further reveal whether adverse selection or advantageous selection prevails among CVD patients. In columns 1–2 of Table 3, we regress the number of GP and ED presentations on only PHI status, while columns 3–4 have included control variables. All estimates from the OLS with and without control variables show that CVD patients with PHI have fewer GP and ED visits compared to those without PHI. Columns 1–2 show that patients with PHI consume 1.89 fewer GP services and have 0.550 fewer ED presentations than those without PHI. These decrease to 1.455 and 0.540, respectively, after including control variables, as in columns 5 and 6. In summary, findings from the OLS support advantageous selection in the uptake of PHI, which is consistent with the results in Table 2.

**TABLE 3:** Private health insurance and the use of primary healthcare services

	OLS without covariates		OLS with covariates	
	GP (1)	ED (2)	GP (3)	ED visits (4)
Private health insurance	−1.891*** (0.076)	−0.550*** (0.023)	−1.455*** (0.074)	−0.540*** (0.023)
Control variables	No	No	Yes	Yes
*N*	175,162	45,694	169,530	45,342

*Note*: All specifications include controls for age, gender, ethnicity, marital status, socioeconomic status, population and unemployment rate, year and SA4 fixed effects. Robust heteroskedasticity standard errors are in parenthesis.

Abbreviations: ED, emergency department; GP, general practitioner; OLS, ordinary least squared.

***p < 0.01, **p < 0.05, *p < 0.1.

### 5.3 Moral hazard

Results for moral hazard in the use of medical services that are covered by PHI providers are presented in Table 4. The estimates from the OLS show that CVD patients with PHI consume more specialist services (0.675), physician services (2.981), miscellaneous diagnostic procedures (0.694) and therapeutic procedures (0.510) than those without PHI. These findings show consistent evidence of *ex‐post* moral hazard among CVD patients. Overall, the largest moral hazard is found for the use of physician services, while the use of therapeutic procedures shows little evidence of moral hazard. No evidence of moral hazard is found in the number of hospital admission between those with and without PHI.^4^

**TABLE 4:** Estimates for moral hazard in healthcare use among CVD patients

	Hospitalizations	Specialist services	Physician services	MDP	TP
	OLS	OLS	OLS	OLS	OLS
Private health insurance	−1.445*** (0.084)	0.675*** (0.029)	2.981*** (0.125)	0.694*** (0.023)	0.510*** (0.043)
SA4 fixed effects	Yes	Yes	Yes	Yes	Yes
Year fixed effects	Yes	Yes	Yes	Yes	Yes
*N*	198,073	169,530	169,530	169,530	169,530

*Note*: All specifications include controls for age, gender, ethnicity, marital status, socioeconomic status, population and unemployment rate, year and SA4 fixed effect. Robust heteroskedasticity standard errors are in parenthesis.

Abbreviations: CVD, cardiovascular disease; MDP, Miscellaneous diagnostic procedures; OLS, ordinary least squared; TP, Therapeutic procedure.

***p < 0.01, **p < 0.05, *p < 0.1.

Our hospital data include information on the nature of hospital presentations; that is, whether they were same‐day, overnight or multiday presentations. We can use this variable to provide more information on the negative correlation between health risk and PHI uptake found by our study. We hypothesize that beyond the fewer GP visits and ED presentations characterizing patients with PHI, such patients are more likely to have more same‐day hospital episodes than their counterparts without PHI. Table 5 reports relative‐risk ratios from a multinomial logit regression with a multiday episode as the reference category. It shows that relative to patients without PHI, those with PHI have a higher risk of same‐day episodes than multiday episodes. Consistently, patients with PHI have a lower risk of experiencing an overnight hospital episode than a multiday episode. These findings suggest that patients with PHI mostly visit the hospital for same‐day procedures while those without PHI go to the hospital for multiday hospital procedures.

**TABLE 5:** Estimates for private health cover and same‐day hospital episodes

Base (multiday episodes)	Same day (1)	Overnight (2)
	RRR	RRR
Private health insurance	2.268*** (0.045)	1.043*** (0.017)
*N*	198,073	198,073

*Note*: All specifications include controls for age, gender, ethnicity, marital status, socioeconomic status, population and unemployment rate, year and SA4 fixed effects. Robust heteroskedasticity standard errors are in parenthesis.

Abbreviation: RRR, relative risk ratio.

***p < 0.01, **p < 0.05, *p < 0.1.

## 6 ADDITIONAL RESULTS AND ROBUSTNESS CHECKS

### 6.1 Private health cover and survival time

**FIGURE 2:**
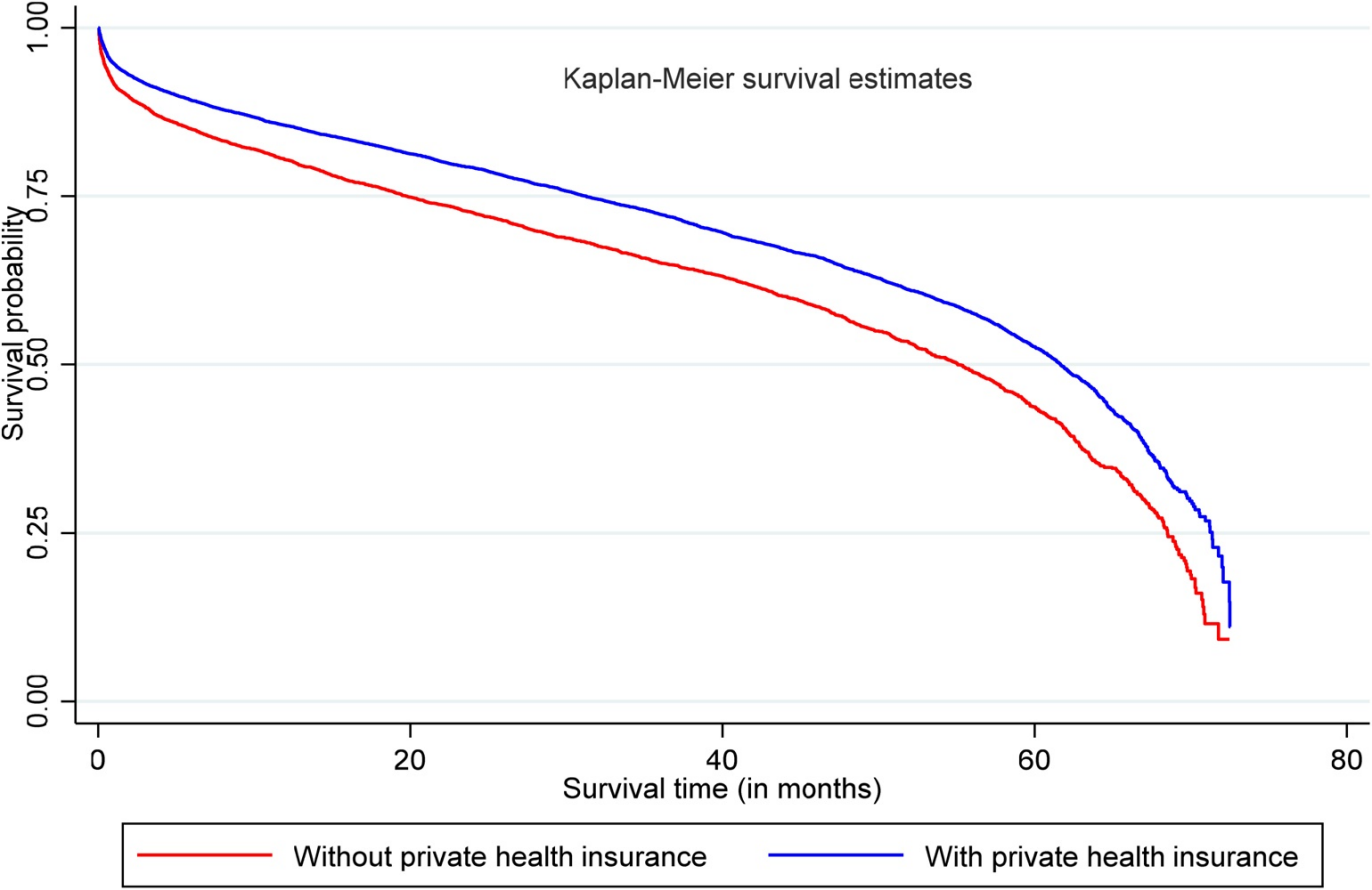
Kaplan‐Meier survival curves for patients with and without private health insurance

We now discuss the differences in the distribution of survival time between patients with and without PHI. This analysis sheds light on the correlation between health risk and PHI uptake. We linked the hospital data with data from the Queensland Registrar of Births, Deaths and Marriages. Survival time is measured as the number of months from the first CVD hospitalization to the date of death. Figure 2 shows that CVD patients with PHI have higher survival probabilities (within 72 months after first‐time CVD hospitalization) than their colleagues without PHI. We further estimate a Cox proportional hazard model and control for all observable patient and geographic characteristics discussed earlier. It is possible that patients with PHI may receive “higher quality” treatment at the hospital, which could affect their survivorship. To isolate this confounding effect, we control for the use of healthcare services that are funded by PHI in our survival modeling. Accordingly, the estimates from the Cox‐proportional hazard model in Table 6 show that patients with PHI have lower odds (11%) of dying compared with those without PHI. These results corroborate the negative correlation between health risk and uptake of PHI (those with PHI have lower odds of dying within 72 months of their first CVD hospitalization).

**TABLE 6:** Estimates for private health cover and survival time

	Time‐to‐death analysis
	HR
Private health insurance	0.890*** (0.024)
*N*	195,213

*Note*: All specifications include controls for age, gender, ethnicity, marital status, socioeconomic status, population and unemployment rate, number of hospitalizations, specialist and physician services used, number of miscellaneous diagnostic procedures, therapeutic procedures, year and SA4 fixed effects. Robust heteroskedasticity standard errors are in parenthesis.

Abbreviation: HR, Hazard ratio.

***p < 0.01, **p < 0.05, *p < 0.1.

### 6.2 Redefining private health insurance ownership status

Since our data are aggregated at the annual level, our proxy for PHI status (only those with PHI at all hospital presentations during the calendar year) can potentially suffer from measurement error because patients who had multiple hospital presentations in a year but had PHI during only one presentation will be captured as not having PHI for the entire calendar year. It is also possible that some patients were admitted as public patients despite having PHI, and their PHI status may not be captured in the hospital admission database.^5^ To gauge the extent of this measurement error, we redefine the exposure status to cover any patients with PHI during at least one hospitalization in a calendar year. Table 7 shows significant evidence of moral hazard in the use of healthcare services, with the largest effect found in the use of physician services. Interestingly, by redefining PHI status to include all patients with PHI during at least one hospitalization, the moral hazard estimates are substantially larger than those in Table 4. This suggests that our benchmark estimates for moral hazard in healthcare use among CVD patients are at the lower bound. This also suggests a substantial change in behavior among CVD patients whenever they obtain PHI cover and thereby, consume more healthcare services.

**TABLE 7:** Estimates for moral hazard after redefining private health cover status

	Hospitalizations	Specialist services	Physician services	MDP	TP
Private health insurance	1.127*** (0.078)	1.141*** (0.023)	7.570*** (0.075)	1.655*** (0.018)	1.110*** (0.229)
*N*	198,073	169,530	169,530	169,530	169,530

*Note*: All specifications include controls for age, gender, ethnicity, marital status, socioeconomic status, population and unemployment rate, year and SA4 fixed effects. Robust heteroskedasticity standard errors are in parenthesis.

Abbreviations: MDP, Miscellaneous diagnostic procedures; TP, therapeutic procedure.

***p < 0.01, **p < 0.05, *p < 0.1.

### 6.3 Cost of moral hazard

We now conduct a cost analysis of moral hazard in the use of healthcare services among our CVD cohort. Specifically, we seek to approximate how much changes in the behavior of the insured cost the PHI provider. Thus, our cost analysis is from the PHI provider's perspective. The cost of healthcare services is obtained from the MBS Online, which is managed by the Australian Government and covers common services in and out of hospitals. We used MBS cost data because they are used as benchmarks for the benefits paid by PHI providers.^6^ Costs are reported in Australian Dollars (A$) and in 2020 values. Unit costs (schedule fees) for specialist and physician attendance are $90.35 (MBS item 104) and $159.35 (MBS item 110), respectively. Similarly, the unit cost of miscellaneous diagnostic procedures is $158.35 (MBS item 11,729), while that of therapeutic procedures is $120.35 (MBS item 13,025). Using the estimates in Table 4 and employing back‐of‐the‐envelope calculations, we estimate that moral hazard among CVD patients costs PHI providers the equivalent of $60.99 (0.675 multiplied by $90.35) and $475.02 (2.981 multiplied by $159.35) per patient per annum in specialist and physician attendance, respectively. The corresponding costs of moral hazard in the use of miscellaneous diagnostic and therapeutic procedures are $109.89 (0.694 multiplied by $158.35) and $61.47 (0.510 multiplied by $120.53), respectively. In total, the cost of moral hazard in the use of healthcare services is $707.34 per CVD patient per annum.^7^ This cost estimate could be a lower bound, as our analysis does not include all the extras covered by PHI providers. Also, our cost estimate does not account for any decrease in the extent of excess service provision if there was less opportunity to obtain excess healthcare services, such as a reduction of tax incentives for PHI.

### 6.4 Use of matching methods

**FIGURE A3:**
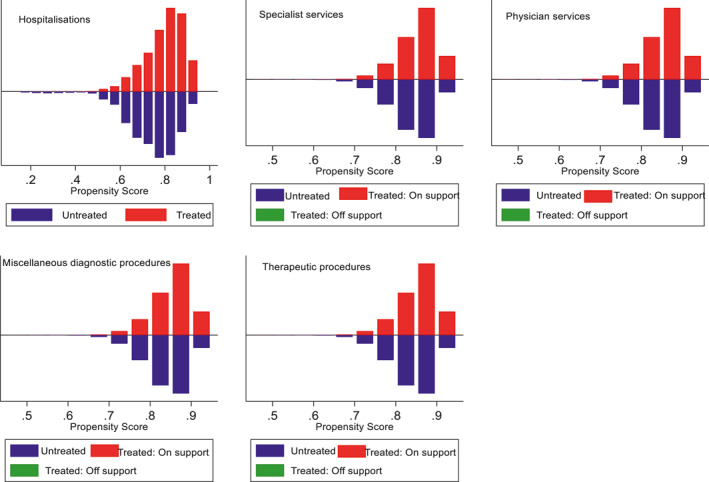
Distribution of propensity score across those with and without private health insurance

Next, we test the robustness of the moral hazard estimates using the propensity score matching approach (PSM). The PSM has gained popularity in estimating causal treatment effects (Brand & Halaby, 2006) due to its ability to address selection bias caused by unobserved heterogeneity. This method has been applied in similar studies to estimate adverse selection and moral hazard in the PHI market (Liu et al., 2012). The PSM can estimate the counterfactual for the treated group (individuals with PHI) by matching individuals with similar propensity scores and then comparing outcomes across treated and control (patients without PHI) groups. We employ the standard Roy‐Rubin framework (Roy, 1951; Rubin, 1973). The PSM resolves selection bias in the uptake of PHI by generating a comparable group based on their propensity scores in the observable characteristics included in the model. Matching was done on the control variables described in Table 1. Figure A3 suggests a high level of common support, since the propensity scores have a similar distribution in the treated (patients with PHI) and comparison groups (patients without PHI). We employ five different matching methods to consider whether the moral hazard estimates are robust. Specifically, we undertake one‐to‐one matching, the 5‐nearest neighbor method, kernel matching, local linear regression, and radius matching approaches.^8^ The average treatment effect on the treated (ATT) results in Table 8 are qualitatively similar to our benchmark estimates for moral hazard in Table 4. That is, irrespective of the matching method used, CVD patients with PHI consume more specialist and physician services, and more miscellaneous diagnostic and therapeutic procedures than those without PHI. Consistently, no evidence of moral hazard is found with regards to the number of hospitalizations among CVD patients.

**TABLE 8:** PSM results from different matching methods

	Hospitalization	Specialist visits	Physician visit	MDP	TP
One‐to‐one	−1.418 (0.908)	0.922*** (0.176)	2.193*** (0.962)	0.677*** (0.197)	0.423 (0.303)
5‐Nearest neighbor	−1.132*** (0.564)	0.838 *** (0.128)	2.300*** (0.789)	0.792*** (0.143)	0.679*** (0.166)
Kernel	−1.486*** (0.449)	0.774*** (0.107)	1.827*** (0.648)	0.697*** (0.119)	0.556*** (0.167)
Local linear regression	−1.271 (0.944)	0.861*** (0.176)	2.475*** (0.961)	0.711*** (0.140)	0.616** (0.303)
Radius	−2.462*** (0.147)	0.728*** (0.047)	1.460*** (0.214)	0.659*** (0.046)	0.461*** (0.074)
*N*	10,253	8754	8754	8754	8754

*Note*: Control variables are those described in Table 1.

Abbreviations: MDP, miscellaneous diagnostic procedures; TP, therapeutic procedures.

***p < 0.01, **p < 0.05, *p < 0.1.

### 6.5 Estimation of selection bias and moral hazard using the conditional mixed‐process estimator

We now estimate moral hazard using the conditional (recursive) mixed process (CMP) estimator. In this approach, Equations (1) and (2) are estimated as a system to consider the possible correlation between their residuals due to individual unobservable characteristics that affect both the decision to purchase PHI and the consumption of health services. The identification strategy of CMP is based on the condition of observability and recursivity as well as on exclusion restriction. Specifically, “observability” means that the dependent variables of interest (e.g., PHI and health service consumption) enter our model as the observed manifest rather than the latent variables underlying them (e.g., utility function), as in Keane and Stavrunova (2016). “Recursivity” means equations in the system (e.g., the PHI and health service equations) can be arranged in a triangular formation where the latent outcome in the current equation is regressed against observed outcomes in previous equations (e.g., Wilde, 2000, eq. 4). Under these two assumptions, Wilde (2000) stated that a multi‐equation probit model is identified if each equation contains at least one varying exogenous regressor. In this case, the varying exogenous regressor can be transformed into multiple binary variables, making the number of independent probabilities larger than the number of unknown parameters (i.e., there are sufficient variations in the data to identify the unknown parameters). Roodman (2011) extended the work of Wilde (2000) to multiple equations with dependent variables of different formats (e.g., binary, categorical, continuous).

Table 9 reports^9^ the result of the CMP approach and shows that moral hazard is still persistent in the use of specialist and physician services, miscellaneous diagnostic procedures, and therapeutic procedures, irrespective of the technique used. The results are qualitatively similar to those from OLS. More importantly, the correlation coefficients (*ρ*) between equations for the uptake of PHI and the of use of healthcare services are statistically insignificant (except that for the use of physician services), suggesting that endogeneity is not compromising the results. Thus, the OLS estimates are robust. Table A4 also reports simultaneous equation results for the decision to purchase PHI; these are similar to those in Table 4.

**TABLE 9:** CMP estimates for moral hazard in the use of healthcare services

	Hospitalizations	Specialist services	Physician services	MDP	TP
	CMP	CMP	CMP	CMP	CMP
Private health insurance	−1.439*** (0.177)	0.678*** (0.305)	3.591*** (0.325)	0.839*** (0.083)	0.634*** (0.126)
*N*	198,073	198,073	198,073	198,073	198,073
*ρ*	−0.00003 (0.008)	0.0003 (0.016)	−0.019** (0.009)	−0.023 (0.013)	−0.011 (0.010)

*Note*: All specifications include controls for age, gender, ethnicity, marital status, socioeconomic status, population and unemployment rate, year and SA4 fixed effects. Robust heteroskedasticity standard errors are in parenthesis.

Abbreviations: CMP, conditional (recursive) mixed process; MDP, miscellaneous diagnostic procedures; TP, therapeutic procedures.

***p < 0.01, **p < 0.05, *p < 0.1.

Although the use of CMP does not require an exclusion (instrumental) variable because of the non‐linear nature of the endogenous variable (PHI) (Roodman, 2011; Wilde, 2000), we test this proposition by including an instrumental variable in Equation (1) and re‐estimate Equations (1) and (2) using the CMP approach. Specifically, we instrument PHI status with the density of PHI across SA4s.^10^ We compute this density variable by removing individuals who have PHI from the overall population for the region in which they reside. The moral hazard estimates in Table A5 are qualitatively and quantitatively similar to those in Table 9. This finding shows that in the CMP framework the use of an exclusion variable is not crucial for identification, as argued by Wilde (2000) and Roodman (2011).

## 7 DISCUSSION AND CONCLUDING REMARKS

Microeconomic theory predicts that insurance markets are characterized by selection bias if insurance providers do not have perfect information about the risk attributes of an individual. That is, high‐risk individuals will be more likely to purchase insurance (adverse selection). Further, due to information asymmetry, the insured are likely to put little effort into preventing a loss or changing their behavior, thereby potentially consuming excessive healthcare services (moral hazard). Selection bias or moral hazard causes market failure by (1) reducing the profit margin of the insurance provider and (2) setting higher/lower premiums for all individuals irrespective of their level of health risk, as well as the possible occurrence of a “death spiral”. This study utilized large longitudinal cohort data of CVD patients to examine the presence of selection bias and moral hazard in the uptake of PHI and the use of healthcare services in a mixed public‐private health system. Our main findings can be summarized as follows.

First, we find robust and consistent evidence of advantageous selection in the uptake of PHI. That is, CVD patients who are younger (below 65 years), have no comorbidities, are married, and of high socioeconomic status are more likely to purchase PHI. Accordingly, such patients use fewer medical services that have unique characteristics; namely, those not covered by PHI (e.g., GP visits) and with shorter waiting times to access (e.g., ED presentations). The findings on the use of GP services are consistent with those found by Nghiem and Graves (2019) and Buchmueller et al. (2013). Also, relative to CVD patients without PHI, those with PHI have higher survival probabilities (less likely to die within 72 months of their first CVD hospitalization), fewer multiple‐day hospital episodes, and fewer comorbidities. These findings show that selection bias is prevalent in the Australian PHI market. However, contrary to the economic hypothesis of adverse selection in the insurance market, our finding reveals advantageous selection: CVD patients with a lower health risk purchase PHI. The presence of advantageous selection among CVD patients in the Australian PHI market indicates that risk‐tolerant CVD individuals enjoy the equilibrium of lower premiums created by the dominant risk‐averse CVD patients.

Second, our findings show evidence of *ex‐post* moral hazard in the use of in‐hospital medical services that are largely funded by PHI providers. These services include specialist and physician services and miscellaneous diagnostic and therapeutic procedures. The largest moral hazard is found in the use of physician services, and it is minimal in the use of miscellaneous therapeutic procedures. Interestingly, we found no evidence of moral hazard in hospital admissions among CVD patients. Rather, patients without PHI had more hospital admissions than those with PHI. One plausible reason for this finding is that although CVD patients with PHI utilize more in‐hospital medical services (e.g., specialist and physician services, and miscellaneous diagnostic and therapeutic procedures), these services are mainly same‐day procedures and do not require admission. This is corroborated by our finding that CVD patients with PHI use more same‐day hospital procedures than their colleagues without PHI. Buchmueller et al. (2013) also found that in Australia, adults with PHI spend fewer nights at the hospital than those without PHI.

Finally, we have estimated that the cost of moral hazard can be substantial to the PHI provider. Specifically, our findings suggest that every year, moral hazard costs the PHI provider approximately $707.34 per CVD patient. Considering that an average of 77.14% of patients had PHI during each year of the study window, this cost translates to approximately $46.2 million per year for insurance providers. To convey the significance of this amount to the PHI provider, we compare this to the June 2021 annual net profit after tax of A$1.5 billion from the PHI industry (Australian Prudential Regulation Authority, 2021). Our findings show that moral hazard in the PHI market reduces the industry's annual profit by 3.08%.^11^ Beyond the impact of moral hazard on the PHI provider, the health system also suffers from such excessive use of healthcare services. With Australia's per capita healthcare expenditure estimated at A$7485 (Australian Institute of Health and Welfare, 2020a), our findings suggest that 9.45% of annual per capita health expenditure can be avoided if the inefficiencies in the PHI market are minimized.

Our findings suggest that tax incentives, such as the MLS, in the Australian PHI market may create inefficiencies. That is, while the MLS encourages high‐income earners, who are potentially low risk, to purchase PHI, there is an *ex‐post* moral hazard once the insurance contract has been activated. From an economic perspective, a Pareto efficient equilibrium might not exist when potentially risk‐tolerant individuals have ample incentives to purchase PHI (Rothschild & Stiglitz, 1976). Also, considering the presence of advantageous selection in the Australian PHI market, a policy that lowers MLS could improve welfare, leading to a strict Pareto efficient outcome (De Meza & Webb, 2001). However, the introduction of a policy lowering the MLS can also reduce the number of people with PHI and hence, is debatable due to the long waiting time for certain elective procedures in public hospitals. Gans and King (2003) recommended the implementation of a reform in the Australian PHI market that regards PHI as a full complementary product to reduce the problem of anti‐insurance. Either way, in as much as the Australian Government aims to increase the uptake of PHI among its populace, this should be done through a cautious and streamlined approach.

## CONFLICT OF INTEREST

We do not have any conflict of interest to declare.

## Data Availability

The data that support the findings of this study are available from Queensland Health. Restrictions apply to the availability of these data, which were used under license for the current study, and so are not publicly available. Data are available from the authors upon reasonable request and with permission of Queensland Health.

## ENDNOTES

^1^
A list of the 19 SA4s in Queensland and their average annual civilian population aged 15 years and above is presented in Table A1.

^2^
Detailed summary statistics are available in Table A2.

^3^
These services are fully covered by Medicare and for GPs who do 100% bulk bill, the patient will not pay any out‐of‐pocket fee. For GPs who do not bulk bill, the patient pays the fees upfront and claim the difference between the rebate and amount charged from government through Medicare.

^4^
These results hold even after separating the data into private versus public hospital admissions.

^5^
Certain exclusions that typically come with health insurance uptake (e.g., waiting periods) can also cause measurement errors in the insurance variable.

^6^
In Australia, private health insurers are required to pay minimum benefit amounts for hospital costs and medical services. However, private health insurers and hospitals often enter into contracts that allow the insurer to pay an amount to the hospital that is higher than the minimum benefit amount. In these situations, the patient would usually not have an out‐of‐pocket cost for hospital care (Australian Medical Association, 2015).

^7^
This figure varies with the MBS item number selected for analysis.

^8^
We randomly selected only 5% of our sample for this analysis because these matching approaches (coupled with our large sample size) take a long time to generate results. Hence, we do so to increase the speed of computation.

^9^
Detailed results are reported in Table A4.

^10^
A measure of the tax incentive for purchasing PHI would be a sound instrumental variable.

^11^
This finding should be interpreted with caution since our moral hazard estimates are from only hospitalized CVD patients in Queensland. Hence, it is potentially a lower bound estimate.

## 1

**TABLE A1:** The 19 Statistical Area Level 4s in Queensland and their average number of civilian members of the population aged 15 years and above for the study period

	Population aged ≥15 years
Brisbane – East	178,066
Brisbane Inner City	165,594
Brisbane – North	276,969
Brisbane – South	146,367
Brisbane – West	209,432
Cairns	186,770
Central Queensland	98,776
Darling Downs – Maranoa	172,666
Gold Coast	446,925
Ipswich	232,998
Logan – Beaudesert	238,819
Mackay ‐ Isaac – Whitsunday	136,709
Moreton Bay – North	182,552
Moreton Bay – South	138,917
Queensland – Outback	64,695
Sunshine Coast	269,344
Toowoomba	115,410
Townsville	175,296
Wide Bay	229,786

**TABLE A2:** Detailed summary statistics of variables used for the analysis

	With private health insurance					Without private health insurance				
	Mean	Median	SD	Min	Max	Mean	Median	SD	Min	Max
GP visits	12.769	10	10.430	0	193	14.379	12	11.518	0	188
ED visits	1.803	1	1.718	1	74	2.353	1	2.809	1	77
Hospital admissions	3.487	2	10.836	1	199	5.394	2	15.789	1	191
Specialist services	2.961	2	4.358	0	183	2.332	1	4.118	0	167
Physician services	9.252	3	17.604	0	249	7.320	2	16.460	0	236
Miscellaneous diagnostic procedure	2.419	1	3.724	0	90	1.782	1	3.436	0	68
Therapeutic procedures	1.207	0	4.913	0	99	0.814	0	3.907	0	89
Male	0.520	1	0.499	0	1	0.546	1	0.497	0	1
Indigenous	0.004	0	0.064	0	1	0.025	0	0.157	0	1
Age groups (years)										
18–44	0.058	0	0.234	0	1	0.071	0	0.258	0	1
45–64	0.286	0	0.451	0	1	0.219	0	0.413	0	1
65 and above	0.656	1	0.475	0	1	0.710	1	0.454	0	1
Socioeconomic status										
Q1	0.127	0	0.332	0	1	0.191	0	0.393	0	1
Q2	0.162	0	0.368	0	1	0.190	0	0.392	0	1
Q3	0.207	0	0.405	0	1	0.200	0	0.400	0	1
Q4	0.223	0	0.416	0	1	0.209	0	0.406	0	1
Q5	0.282	0	0.449	0	1	0.211	0	0.409	0	1
Marital status										
Divorced	0.057	0	0.232	0	1	0.070	0	0.255	0	1
Married/de facto	0.685	1	0.464	0	1	0.511	1	0.499	0	1
Never married	0.089	0	0.286	0	1	0.138	0	0.345	0	1
Widowed	0.167	0	0.373	0	1	0.280	0	0.449	0	1
Comorbidity status										
No	0.454	0	0.497	0	1	0.318	1	0.465	0	1
One	0.137	0	0.343	0	1	0.134	0	0.341	0	1
Two or more	0.408	0	0.491	0	1	0.548	1	0.498	0	1

**TABLE A3:** Average transition probabilities into private cover status (%)

To		No	Yes
From	No	60.08	39.92
	Yes	8.0	92

*Note*: xttrans command in STATA was used to estimate transition probabilities.

**TABLE A4:** CMP estimates for selection bias and moral hazard in private health insurance

	Private health insurance		Hospitalization	Specialist services	Physician services	MDP	TP
	Coefficients	Marginal effects	Coefficients	Coefficients	Coefficients	Coefficients	Coefficients
Private health insurance	N/A	N/A	−1.439*** (0.177)	0.678*** (0.305)	3.591*** (0.325)	0.839*** (0.083)	0.634*** (0.126)
Male	−0.170*** (0.007)	−0.046*** (0.002)	0.148*** (0.055)	−0.096*** (0.022)	−0.509*** (0.091)	0.338** (0.018)	0.278*** (0.033)
Indigenous	−0.952*** (0.033)	−0.261*** (0.009)	3.483*** (0.620)	0.689*** (0.148)	−0.230 (0.605)	0.045 (0.121)	−0.367* (0.219)
Age (ref = 18–44)							
45–64	0.295*** (0.015)	0.082*** (0.004)	−0.347*** (0.120)	0.235*** (0.047)	0.748*** (0.192)	0.624*** (0.038)	0.202*** (0.070)
65 and above	0.241*** (0.015)	0.067*** (0.004)	−0.771*** (0.123)	0.834*** (0.048)	2.864*** (0.191)	1.474*** (0.038)	0.135* (0.069)
Marital status (ref = divorced)							
Married	0.327*** (0.013)	0.089*** (0.004)	0.583*** (0.099)	0.362*** (0.045)	0.626*** (0.184)	0.322*** (0.037)	0.228*** (0.067)
Never married	−0.050*** (0.016)	−0.014*** (0.004)	1.700*** (0.147)	0.436*** (0.055)	2.434*** (0.224)	0.262*** (0.044)	0.452*** (0.081)
Widowed	−0.177*** (0.015)	−0.049*** (0.004)	0.187 (0.119)	0.151*** (0.053)	3.127*** (0.213)	0.488*** (0.042)	−0.109 (0.077)
SES (ref = Q1)							
Q2	0.131*** (0.015)	0.036*** (0.004)	0.393*** (0.131)	−0.048 (0.053)	0.940*** (0.215)	0.046 (0.043)	0.100 (0.078)
Q3	0.288*** (0.015)	0.079*** (0.004)	0.857*** (0.134)	0.163*** (0.053)	1.235*** (0.216)	−0.045 (0.043)	0.206*** (0.078)
Q4	0.280*** (0.016)	0.077*** (0.004)	0.820*** (0.142)	0.110** (0.056)	1.689*** (0.228)	0.065 (0.045)	0.247*** (0.083)
Q5	0.368*** (0.017)	0.100*** (0.005)	0.929*** (0.149)	0.162*** (0.058)	2.279*** (0.238)	0.040 (0.047)	0.248*** (0.086)
Comorbidity (ref = none)							
One	−0.143*** (0.010)	−0.039*** (0.003)	0.757*** (0.029)	0.247*** (0.034)	3.994*** (0.138)	0.967*** (0.027)	0.676*** (0.050)
Two or more	−0.309*** (0.008)	−0.085*** (0.003)	4.415*** (0.066)	0.997*** (0.025)	11.821*** (0.103)	1.033*** (0.021)	1.627*** (0.037)
Unemployment rate	−0.001 (0.002)	−0.0001 (0.001)	−0.028** (0.013)	−0.025*** (0.008)	−0.077** (0.033)	−0.028*** (0.007)	−0.010 (0.012)
Log (population)	1.929*** (0.126)	0.529*** (0.035)	15.647*** (1.012)	1.291*** (0.416)	−13.425*** (1.688)	−5.156*** (0.334)	−8.070*** (0.611)
Year fixed effects	Yes	Yes	Yes	Yes	Yes	Yes	Yes
SA4 fixed effects	Yes	Yes	Yes	Yes	Yes	Yes	Yes
*N*	198,073		198,073	198,073	198,073	198,073	198,073
*ρ*	N/A		−0.00003 (0.008)	0.0003 (0.016)	−0.019** (0.009)	−0.023 (0.013)	−0.011 (0.010)

*Note*: Robust standard errors are in parentheses. We have reported only one results for the determinants of private health insurance uptake from the CMP models because the results are similar across all specifications.

Abbreviations: CMP, conditional (recursive) mixed process; MDP, miscellaneous diagnostic procedures; SES, Socioeconomic status; TP, therapeutic procedures.

****p* < 0.01, ***p* < 0.05, **p* < 0.1.

**TABLE A5:** CMP estimates for moral hazard in the use of healthcare services using insurance density as an instrumental variable

	Hospitalizations	Specialist services	Physician services	MDP	TP
Private health insurance	−1.424*** (0.095)	0.673*** (0.060)	3.611*** (0.164)	0.818*** (0.038)	0.610*** (0.063)
First stage (instrument)					
Insurance density	2.856*** (0.287)	2.852*** (0.287)	2.860*** (0.287)	2.845*** (0.287)	2.851*** (0.287)
*N*	198,073	198,073	198,073	198,073	198,073
*ρ*	−0.0008 (0.001)	0.001 (0.006)	−0.020*** (0.002)	−0.020*** (0.003)	−0.010*** (0.003)

*Note*: All specifications include controls for age, gender, ethnicity, marital status, socioeconomic status, population and unemployment rate, year and SA4 fixed effects. Robust heteroskedasticity standard errors are in parenthesis.

Abbreviations: CMP, conditional (recursive) mixed process; MDP, miscellaneous diagnostic procedures; TP, therapeutic procedures.

****p* < 0.01, ***p* < 0.05, **p* < 0.1.

## References

[hec4605-bib-0001] Afoakwah, C. , Nghiem, S. , Scuffham, P. , & Byrnes, J. (2021). Rising unemployment reduces the demand for healthcare services among people with cardiovscular disease: An Australian cohort study. The European Journal of Health Economics, 22(4), 643–658. 10.1007/s10198-021-01281-5 33740154

[hec4605-bib-0054] Afoakwah, C. , Nghiem, S. , Scuffham, P. , Huynh, Q. , Marwick, T. , & Byrnes, J. (2020). Impacts of air pollution on health: evidence from longitudinal cohort data of patients with cardiovascular diseases. The European Journal of Health Economics, 21(7), 1025–1038. 32415421 10.1007/s10198-020-01198-5

[hec4605-bib-0002] Akerlof, G. A. (1970). The market for “lemons”: Quality uncertainty and the market mechanism. Quarterly Journal of Economics, 84(3), 488–500. 10.2307/1879431

[hec4605-bib-0003] Alam, M. R. , Kabir, M. R. , & Reza, S. (2021). Comorbidities might be a risk factor for the incidence of COVID‐19: Evidence from a web‐based survey. Journal of Preventive Medicine Reports, 21, 101319. 10.1016/j.pmedr.2021.101319 33489728 PMC7811036

[hec4605-bib-0004] Arrow, K. (1963). Uncertainty and the welfare economics of medicare care. The American Economic Review, 53, 941–973.

[hec4605-bib-0005] Australian Bureau of Statistics . (2021). National, state and territory population. https://www.abs.gov.au/statistics/people/population/national‐state‐and‐territory‐population/latest‐release

[hec4605-bib-0007] Australian Government Department of Health and Aged Care . (2021). About private health insurance. https://www.health.gov.au/health‐topics/private‐health‐insurance/about‐private‐health‐insurance

[hec4605-bib-0006] Australian Government Department of Health . (2019). What private health insurance covers. https://www.health.gov.au/health‐topics/private‐health‐insurance/what‐private‐health‐insurance‐covers

[hec4605-bib-0008] Australian Institute of Health and Welfare . (2020a). Health expenditure. https://www.aihw.gov.au/reports/australias‐health/health‐expenditure

[hec4605-bib-0009] Australian Institute of Health and Welfare . (2020b). Private health insurance. https://www.aihw.gov.au/reports/australias‐health/private‐health‐insurance

[hec4605-bib-0010] Australian Medical Association . (2015). Guide for patients on how the health care system funds medical care. Retrieved September 13, 2021 https://www.ama.com.au/articles/guide‐patients‐how‐health‐care‐system‐funds‐medical‐care

[hec4605-bib-0011] Australian Prudential Regulation Authority . (2021). APRA releases quarterly private health insurance statistics for June 2021. https://www.apra.gov.au/news‐and‐publications/apra‐releases‐quarterly‐private‐health‐insurance‐statistics‐for‐june‐2021

[hec4605-bib-0012] Australian Prudential Regulatory Authority . (2021). Quarterly private health insurance statistics – highlights. https://www.apra.gov.au/sites/default/files/2021‐05/Quarterly%20private%20health%20insurance%20statistics%20highlights%20March%202021.pdf

[hec4605-bib-0013] Barrett, G. F. , & Conlon, R. (2003). Adverse selection and the decline in private health insurance coverage in Australia: 1989–95. The Economic Record, 79(246), 279–296. 10.1111/1475-4932.00104

[hec4605-bib-0014] Bolhaar, J. , Lindeboom, M. , & Van Der Klaauw, B. (2012). A dynamic analysis of the demand for health insurance and health care. European Economic Review, 56(4), 669–690. 10.1016/j.euroecorev.2012.03.002

[hec4605-bib-0015] Brand, J. E. , & Halaby, C. N. (2006). Regression and matching estimates of the effects of elite college attendance on educational and career achievement. Social Science Research, 35(3), 749–770. 10.1016/j.ssresearch.2005.06.006

[hec4605-bib-0016] Buchmueller, T. C. , Fiebig, D. G. , Jones, G. , & Savage, E. (2013). Preference heterogeneity and selection in private health insurance: The case of Australia. Journal of Health Economics, 32(5), 757–767. 10.1016/j.jhealeco.2013.05.001 23770762

[hec4605-bib-0017] BUPA . (2022). Why get private health insurance. https://www.bupa.com.au/health‐insurance/why‐choose‐bupa/why‐get‐private‐health‐insurance

[hec4605-bib-0018] Byrnes, J. , Nghiem, S. , Afoakwah, C. , & Scuffham, P. (2020). Queensland cardiovascular data linkage (QCard): A population‐based cohort study [version 1; peer review: Awaiting peer review]. F1000Research, 9(282). 10.12688/f1000research.23261.1

[hec4605-bib-0019] Cameron, A. C. , & Trivedi, P. K. (2005). Microeconometrics: Methods and applications. Cambridge university press.

[hec4605-bib-0020] Cawley, J. , & Philipson, T. (1999). An empirical examination of information barriers to trade in insurance. The American Economic Review, 89(4), 827–846. 10.1257/aer.89.4.827

[hec4605-bib-0022] Chiappori, P. , & Salanie, B. (2000). Testing for asymmetric information in insurance markets. Journal of Political Economy, 108(1), 56–78. 10.1086/262111

[hec4605-bib-0021] Chiappori, P. , Jullien, B. , Salanié, B. , & Salanie, F. (2006). Asymmetric information in insurance: General testable implications. The RAND Journal of Economics, 37(4), 783–798. 10.1111/j.1756-2171.2006.tb00057.x

[hec4605-bib-0023] d e Meza, D. , & Webb, D. C. (2001). Advantageous selection in insurance markets. The RAND Journal of Economics, 32(2), 249–262. 10.2307/2696408

[hec4605-bib-0024] Dionne, G. , Gouriéroux, C. , & Vanasse, C. (2001). Testing for evidence of adverse selection in the automobile insurance market: A comment. Journal of Political Economy, 109(2), 444–453. 10.1086/319557

[hec4605-bib-0025] Doiron, D. , Fiebig, D. G. , & Suziedelyte, A. (2013). Hips and hearts: The variation in moral hazard across hospital procedures. 10.1016/j.jhealeco.2014.06.006 24981504

[hec4605-bib-0026] Eldridge, D. S. , Onur, I. , & Velamuri, M. (2017). The impact of private hospital insurance on the utilization of hospital care in Australia. Applied Economics, 49(1), 78–95. 10.1080/00036846.2016.1192273

[hec4605-bib-0027] Fang, H. , Keane, M. P. , & Silverman, D. (2008). Sources of advantageous selection: Evidence from the Medigap insurance market. Journal of Political Economy, 116(2), 303–350. 10.1086/587623

[hec4605-bib-0028] Finkelstein, A. , & McGarry, K. (2006). Multiple dimensions of private information: Evidence from the long‐term care insurance market. The American Economic Review, 96(4), 938–958. 10.1257/000282806779468427 29135205

[hec4605-bib-0029] Finkelstein, A. , & Poterba, J. (2002). Selection effects in the United Kingdom individual annuities market. The Economic Journal, 112(476), 28–50. 10.1111/1468-0297.0j672

[hec4605-bib-0030] Finkelstein, A. , & Poterba, J. (2004). Adverse selection in insurance markets: Policyholder evidence from the UK annuity market. Journal of Political Economy, 112(1), 183–208. 10.1086/379936

[hec4605-bib-0031] Gans, J. S. , & King, S. P. (2003). Anti‐insurance: Analysing the health insurance system in Australia. The Economic Record, 79(247), 473–486. 10.1111/j.1475-4932.2003.00146.x

[hec4605-bib-0032] Hemenway, D. (1990). Propitious selection. Quarterly Journal of Economics, 105(4), 1063–1069. 10.2307/2937886

[hec4605-bib-0033] Hemenway, D. (1992). Propitious selection in insurance. Journal of Risk and Uncertainty, 5(3), 247–251. 10.1007/bf00057881

[hec4605-bib-0034] Keane, M. , & Stavrunova, O. (2016). Adverse selection, moral hazard and the demand for Medigap insurance. Journal of Econometrics, 190(1), 62–78. 10.1016/j.jeconom.2015.08.002

[hec4605-bib-0035] Liu, X. , Nestic, D. , & Vukina, T. (2012). Estimating adverse selection and moral hazard effects with hospital invoices data in a government‐controlled healthcare system. Health Economics, 21(8), 883–901. 10.1002/hec.1756 21648014

[hec4605-bib-0036] Markham, D. , & Graudins, A. (2011). Characteristics of frequent emergency department presenters to an Australian emergency medicine network. BMC Emergency Medicine, 11(1), 1–5. 10.1186/1471-227x-11-21 22171720 PMC3267650

[hec4605-bib-0037] McFadden, D. (1974). Conditional logit analysis of qualitative choice behavior. In P. Zarembka (Ed.), Frontiers in econometrics (pp. 104–142). Academic Press.

[hec4605-bib-0038] Mitchell, O. S. , & McCarthy, D. (2002). Estimating international adverse selection in annuities. North American Actuarial Journal, 6(4), 38–54. 10.1080/10920277.2002.10596062

[hec4605-bib-0039] Nghiem, S. , & Graves, N. (2019). Selection bias and moral hazard in the Australian private health insurance market: Evidence from the Queensland skin cancer database. Economic Analysis and Policy, 64, 259–265. 10.1016/j.eap.2019.09.008

[hec4605-bib-0040] Nguyen, L. , & Worthington, A. (2017). Adverse selection in private health insurance. Consumer Interests Annual, 63, 25–34.

[hec4605-bib-0042] Pauly, M. V. (1974). Overinsurance and public provision of insurance: The roles of moral hazard and adverse selection. Quarterly Journal of Economics, 88(1), 44–54. 10.2307/1881793

[hec4605-bib-0041] Pauly , M. V. (1968). The economics of moral hazard: Comment. The American Economic Review, 58(3), 531–537.

[hec4605-bib-0043] Roodman, D. (2011). Fitting fully observed recursive mixed‐process models with CMP. STATA Journal, 11(2), 159–206. 10.1177/1536867x1101100202

[hec4605-bib-0044] Rothschild, M. , & Stiglitz, J. (1976). Equilibrium in competitive insurance markets: An essay on the economics of imperfect information. Quarterly Journal of Economics, 90(4), 629–649. 10.2307/1885326

[hec4605-bib-0045] Roy, A. D. (1951). Some thoughts on the distribution of earnings. Oxford Economic Papers, 3(2), 135–146. 10.1093/oxfordjournals.oep.a041827

[hec4605-bib-0046] Rubin, D. (1973). The use of matched sampling and regression adjustment to remove bias in observational studies (pp. 185–203). Biometrics.

[hec4605-bib-0047] Saunders, C. (2018). Economic cost of acute coronary syndrome in Australia: The cost to governments. National Heart Foundation. https://resources.heartfoundation.org.au/images/uploads/publications/ACS_Overview_of_Reports_‐_Cost_of_ACS_to_Governments__Individuals_and_Families_August_2018.pdf

[hec4605-bib-0048] Savage, E. , & Wright, D. (2003). Moral hazard and adverse selection in Australian private hospitals: 1989–1990. Journal of Health Economics, 22(3), 331–359. 10.1016/s0167-6296(02)00104-2 12683956

[hec4605-bib-0049] Spence, M. , & Zeckhauser, R. (1971). Insurance, information, and individual action. The American Economic Review, 61(2), 380–387.

[hec4605-bib-0050] van Gool, K. , Parkinson, B. , & Kenny, P. (2015). Medicare Australia data for research: An introduction. https://www.uts.edu.au/sites/default/files/2019‐04/crest‐factsheet‐medicare‐australia.pdf

[hec4605-bib-0051] White, H. (1980). A heteroskedasticity‐consistent covariance matrix estimator and a direct test for heteroskedasticity. Econometrica, 48(4), 817–838. 10.2307/1912934

[hec4605-bib-0052] Wilde, J. (2000). Identification of multiple equation probit models with endogenous dummy regressors. Economics Letters, 69(3), 309–312. 10.1016/s0165-1765(00)00320-7

